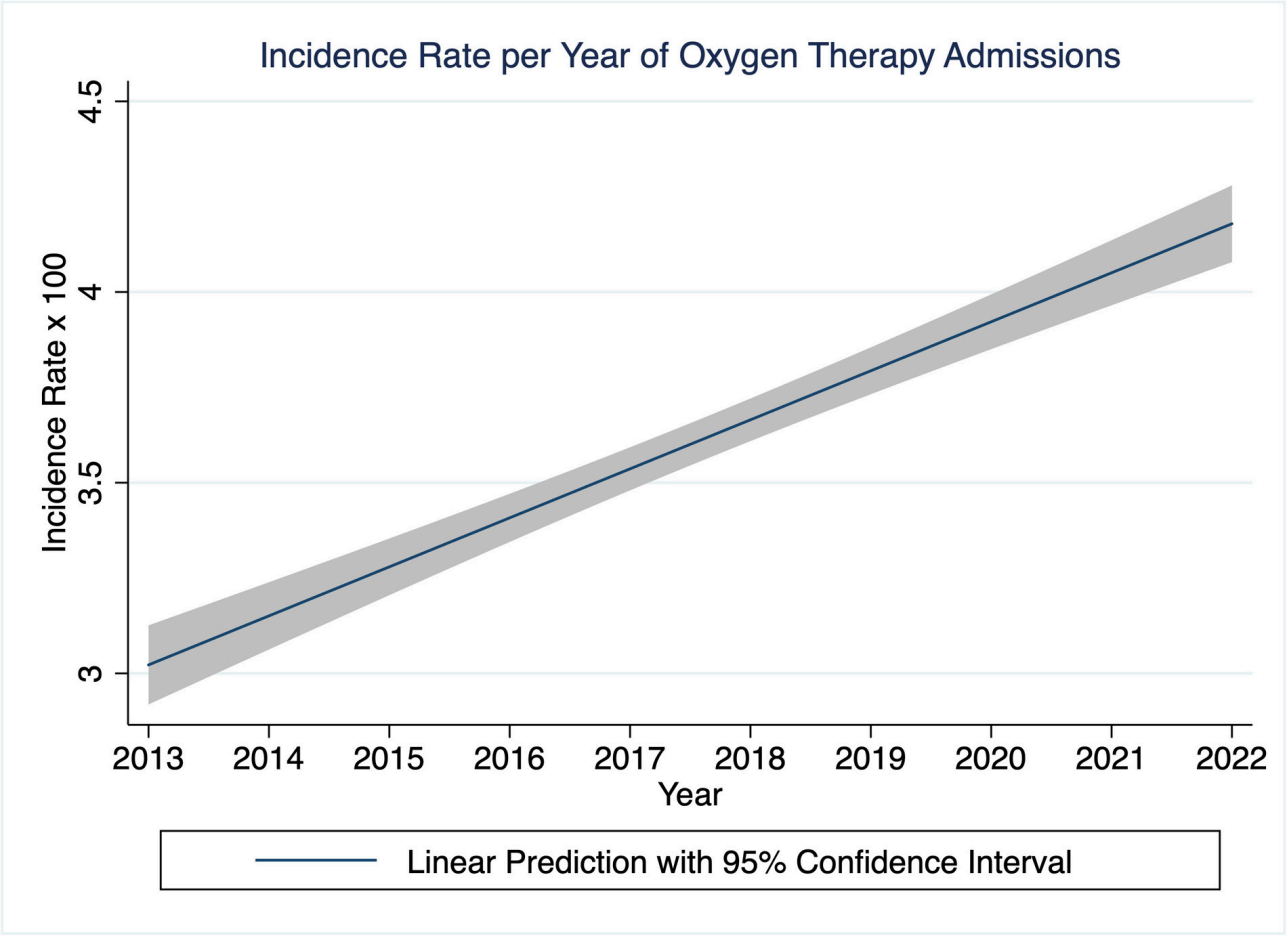# 649 National Estimates and Outcomes for Oxygen Therapy Injuries—a BCQP Analysis

**DOI:** 10.1093/jbcr/iraf019.278

**Published:** 2025-04-01

**Authors:** Clifford Sheckter, Bart Phillips, Karla Klas, Rebecca Coffey, Alisa Savetamal, Lucy Wibbenmeyer

**Affiliations:** Stanford University; BData, Inc; University of Michigan, Michigan Medicine Trauma Burn Center; Parkland Burn Center; Yale University; University of Iowa

## Abstract

**Introduction:**

Current estimates of home oxygen related burns, inhalation injury, and death may be significantly lower than actual incidence. This is problematic in scoping the issue as the American Burn Association continues its work in preventing home oxygen related fire injuries. A lack of diagnosis/injury codes precludes accurate estimates from national databases. The Burn Care Quality Platform (BCQP) database has a free text field for injury description which is optional to complete for burn centers. We leveraged this data source to generate national estimates of home oxygen therapy injuries and outcomes.

**Methods:**

BCQP was queried from 2013-2022 to characterize patients admitted with oxygen therapy injuries. Text string extraction including multiple permutations of words including supplemental/home oxygen was performed in the injury description field of BCQP to identify encounters. Patient demographics, injury characteristics, and inpatient outcomes were described. A logistic regression evaluated predictors of oxygen therapy injuries based on key covariates including white race, age, gender, h/o chronic obstructive pulmonary disease, smoker, chronic respiratory illness, burn size, and smoke inhalation injury. This model was used to predict oxygen therapy injures in the non-responder cohort. Poisson regression estimated adjusted incidence rates.

**Results:**

Of the 207,364 admissions, 28% (79,114) had injury descriptions of which 3.0% were due to oxygen therapy. Oxygen therapy admissions were: 65 years median age (IQR 59, 71), 1.5% median TBSA (IQR 1, 3), 62.6% male, 79.0% white race, 11.4% with smoke inhalation injury, 3 days median LOS (IQR 1, 9), 57.7% with Medicare as primary payer, and 6.1% inpatient mortality. Burn center admissions (72%) that did not report injury text fields admitted fewer smoke inhalation injuries, smaller burns, younger patients, and fewer white patients. A logistic model demonstrated a 0.93 area under curve prediction for home oxygen injury in the responder cohort. The adjusted incidence rate of home oxygen injuries was 3.6% (95% CI 2.7-2.9%, p< 0.001). There was a significant increase in oxygen therapy admissions over the study period (incidence rate ratio 1.03, 95% CI 1.02-1.04). The 2022 US population-adjusted incidence of home oxygen therapy injuries admitted to burn centers was 1,088/year (95% CI 1,065-1,113) or 6.9/100,000 COPD patients across the US.

**Conclusions:**

Oxygen therapy injuries represent 3.6% of all burn admissions in the US and are increasing. Despite being smaller burns, mortality is two times higher the national average for a US burn patient.

**Applicability of Research to Practice:**

Burn centers should contribute to the injury text field in BCQP to capture details not included in ICD-10-CM coding. Oxygen therapy fires are common, increasing, and deadly. Ongoing prevention efforts are needed.

**Funding for the Study:**

N/A